# Clinical backgrounds and outcomes of patients with Barrett's esophageal adenocarcinoma treated via endoscopic submucosal dissection in Kyushu, Japan: A retrospective multicenter cohort study

**DOI:** 10.1002/deo2.70102

**Published:** 2025-05-05

**Authors:** Fumisato Sasaki, Kazuhiro Mizukami, Taro Akashi, Naoyuki Yamaguchi, Ryosuke Gushima, Hideaki Miyamoto, Shohei Uehara, Yoichiro Ono, Takashi Hisabe, Yuzuru Kinjo, Yuichiro Nasu, Kensuke Fukuda, Taisuke Inada, Yorinobu Sumida, Takashi Akutagawa, Tadashi Miike, Sho Suzuki, Hiroki Fukuya, Michita Mukasa, Mitsuru Esaki, Shinsuke Kumei, Yosuke Minoda, Tetsu Kinjo, Daisuke Yamaguchi, Yoshio Fukuda, Kazutoshi Hashiguchi, Hiroki Yano, Hiroshi Fujita, Ryo Shimoda, GI‐Kysuhu study group

**Affiliations:** ^1^ Digestive and Lifestyle Diseases Kagoshima University Graduate School of Medical and Dental Sciences Kagoshima Japan; ^2^ Department of Gastroenterology Oita University Oita Japan; ^3^ Department of Gastroenterology and Hepatology Nagasaki University Graduate School of Biomedical Sciences Nagasaki Japan; ^4^ Department of Gastroenterology and Hepatology Faculty of Life Sciences Kumamoto University Kumamoto Japan; ^5^ Department of Gastroenterology Fukuoka University Chikushi Hospital Fukuoka Japan; ^6^ Department of Gastroenterology Naha City Hospital Okinawa Japan; ^7^ Division of Gastroenterology Kagoshima City Hospital Kagoshima Japan; ^8^ Department of Gastroenterology Kitakyushu Municipal Medical Center Fukuoka Japan; ^9^ Department of Endoscopic Diagnostics and Therapeutics Saga University Hospital Saga Japan; ^10^ Department of Internal Medicine Division of Gastroenterology and Hepatology Faculty of Medicine University of Miyazaki Miyazaki Japan; ^11^ Department of Gastroenterology Clinical Research Institute National Hospital Organization Kyushu Medical Center Fukuoka Japan; ^12^ Department of Medicine Division of Gastroenterology Kurume University School of Medicine Fukuoka Japan; ^13^ Department of Gastroenterology Harasanshin Hospital Fukuoka Japan; ^14^ Third Department of Internal Medicine School of Medicine University of Occupational and Environmental Health Fukuoka Japan; ^15^ Department of Medicine and Bioregulatory Science Graduate School of Medical Sciences Kyushu University Fukuoka Japan; ^16^ Department of Endoscopy University of the Ryukyus Hospital Okinawa Japan; ^17^ Department of Gastroenterology National Hospital Organization Ureshino Medical Center Saga Japan; ^18^ Department of Internal Medicine Division of Gastroenterology Faculty of Medicine Saga University Saga Japan; ^19^ Division of Gastroenterology Kagoshima Kouseiren Hospital Kagoshima Japan; ^20^ Department of Gastroenterology Imamura Hospital Saga Japan; ^21^ Division of Gastroenterology Oshima Prefectural Hospital Kagoshima Japan; ^22^ Division of Gastroenterology Izumi General Medical Center Kagoshima Japan

**Keywords:** adenocarcinoma, Barrett's esophagus, cohort study, endoscopic submucosal dissection, esophageal squamous cell carcinoma

## Abstract

**Objectives:**

Most esophageal cancers in Japan are squamous cell carcinomas; however, there has been some concern regarding a recent increase in Barrett's esophageal adenocarcinoma (BEA). This study aimed to clarify the clinical characteristics and outcomes of patients treated via endoscopic submucosal dissection (ESD) in Kyushu, including changes over time.

**Methods:**

This multicenter, retrospective, observational study was conducted among 21 institutes situated in Kyushu. Data from patients who underwent ESD for BEA or esophageal squamous cell carcinoma between January 2010 and December 2023 were extracted from the pathology database and reviewed.

**Results:**

The total number of esophageal ESD cases increased from 2299 over the first 7 years to 4009 over the second seven. The incidence of BEA increased from 3.6% (86/2299) over the earlier period to 4.7% (197/4009; *p* = 0.034) over the latter. We analyzed data from 283 patients (287 lesions). Smaller tumor‐sized lesions were detected over the latter period (14.2 ± 11.6 vs. 11.2 ± 9.5 cm^2^, *p* = 0.022), significantly reducing treatment times (122.1 ± 81.2 vs. 93.2 ± 53.3 min *p* < 0.001). The procedure was safe, with low incidence rates, over both the earlier and later periods (respectively), of perforation (0% vs. 1.0%), delayed bleeding (1.2% vs. 2.0%), and pneumonia (4.7% vs. 4.6%).

**Conclusion:**

The proportion of esophageal ESD procedures to treat BEA has increased in Japan's Kyushu region. This procedure has a comparable safety profile to similar ESD procedures used to treat other conditions.

## INTRODUCTION

The development of endoscopic techniques such as image enhancement endoscopy (IEE) has led to increased detection of early‐stage gastrointestinal cancers.^1^, ^2^, ^3^, ^4^ Endoscopic submucosal dissection (ESD) has been widely used to treat such cancers, with generally favorable results.^5^, ^6^, ^7^ Most esophageal cancers in Japan are squamous cell carcinomas (SCCs); however, concerns have been rising in recent years regarding Barrett's esophageal adenocarcinoma (BEA) and gastroesophageal reflux disease (GERD).^8^, ^9^

This shift may be because of the recent increase in obesity in the country, owing to changing dietary habits; higher incidence rates of hiatus hernia, reflux esophagitis, and gastric acid secretion; and lower *Helicobacter pylori* (Hp) infection rates.^8^, ^9^ Higher incidence rates of both Barrett's esophagus (BE)^9^, ^10^ and BEA^11^, ^12^ have been reported in Japan over recent years. The recent development of IEE has led to more detection of many early‐stage gastrointestinal cancers, including esophageal SCC.^1^ Previous reports concerning the increased number of BE cases have mainly been based on cancer registry databases; thus, it remains unclear whether the increase is attributable to evolving trends in patient‐related factors or the development of endoscopic techniques such as IEE and the widespread adoption of ESD. It also remains difficult to determine whether advanced esophagogastric junction (EGJ) adenocarcinomas originate from the esophagus or stomach. This study evaluated the proportion of BEA cases among those of overall esophageal ESD, including temporal shifts that may accurately reflect the increased BEA prevalence that has recently been observed.

Japan's geriatric population continues to increase and is expected to peak by 2040.^13^ The population of the Kyushu region has one of the highest median ages in the country and includes several areas with high incidence rates of esophageal SCC.^14^ As the incidence of esophageal SCC increases with age,^15^ it is likely increasing in Kyushu as well. Conversely, BEA's age of onset has been reported to be earlier than that of esophageal SCC.^16^ Investigating BEA trends in Kyushu's older population may therefore help predict future trends regarding malignant esophageal diseases throughout the rest of Japan.

This study aimed to clarify trends in the prevalence, clinical characteristics, and treatment outcomes of patients with BEA who were treated via ESD in Kyushu, as well as the associated temporal trends.

## METHODS

### Study design and patients

This multicenter, retrospective, observational study was conducted across 21 institutions in Kyushu, Japan (the GI‐Kyushu Study Group), including 11 university hospitals and 10 general hospitals. The records of all patients who underwent ESD for BEA or esophageal SCC between January 2010 and December 2023 were extracted from the pathology database and reviewed.

Cases of BEA treated via endoscopic mucosal resection (EMR) for BEA were excluded. This is because EMR may not provide accurate assessments of the double muscularis and submucosal esophageal glands, which comprise the necessary pathological evidence for diagnosing BEA.

**FIGURE 1:**
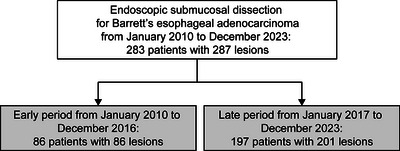
Flow chart of the processes for patient enrolment and analysis of the lesions.

The enrolled patients met the following criteria:

BEA patients: (1) histologically diagnosed esophageal adenocarcinoma, (2) lesions that were endoscopically confirmed to have occurred within or near the BE area, and (3) age ≥20 years old at the time of ESD. A total of 283 patients (287 lesions) were included, and divided into groups according to their procedure date—with January 2010 to December 2016 forming the early period and January 2017 to December 2023 comprising the late period (Figure 1).

SCC patients: (1) histologically diagnosed SCC, (2) age ≥20 years old at the time of ESD, and (3) SCC patients treated with ESD between April 2010 and December 2023.‘’

### Primary endpoint

The primary study endpoint was temporal trends concerning the number, clinical characteristics, and treatment outcomes of patients with BEA who were treated via ESD over the study period. Thus, the proportion of BEA among the total number of esophageal ESD cases (including ones of SCC) that underwent ESD were retrospectively compared.

### Outcomes

Patient characteristics such as background (age, sex, body mass index [BMI] at diagnosis, drinking history, smoking history, prior cancer history in other organs, use of antithrombotic drugs and proton pump inhibitors, and presence of Hp infection), BE length (short segment BE or long segment BE), presence and severity of reflux esophagitis and hiatus hernia, lesion characteristics (tumor location, macroscopic type, depth, and diameter), and patient outcomes (en bloc resection rate, cure resection rate, resection length, resection time, dissection speed, and incidence of complications) were examined retrospectively.

### Parameter definitions

1. Hp: Serum IgG antibody, urea breath, stool antigen, and urinary antibody tests were performed to test for Hp, and patients with any positive test results were considered positive.^17^ Hp‐uninfected patients were defined as those with: 1) no history of Hp eradication, RAC‐positive endoscopic findings, and negative results for one Hp test; or 2) negative results for at least two Hp tests. Patients with no history of Hp eradication and positive endoscopic findings for RAC were defined as having suspected Hp infection.
2. BE and EGJ: Endoscopic BE was defined as a columnar‐lined distal esophagus between the squamous columnar junction and EGJ. The endoscopic EGJ was defined as the lower margin of the palisading small vessels, according to the criteria proposed by the Japan Esophageal Society.^18^ If the small palisading vessels were unclear, the EGJ was defined as the oral margin of the longitudinal folds of the greater curvature of the stomach folds. Short segment BE was defined as cases wherein Barrett's mucosa was <3 cm or non‐peripheral, while long segment BE was defined as cases wherein it was ≥3 cm in circumferential extent.^19^, ^20^
3. Reflux esophagitis and hiatal hernia: The severity of reflux esophagitis was determined using the revised Los Angeles classification.^21^ The presence or absence of a hiatal hernia was confirmed using endoscopic findings and noted.^22^
4. ESD treatment outcomes: The rate of *en bloc* resection was defined as the number of cases where *en bloc* resection was possible endoscopically. Curative resection was defined as endoscopic *en bloc* resection with pT1a, deep muscularis mucosa (DMM), negative horizontal or vertical margins, and no lympho‐vascular invasion.^19^ Procedure time was defined as the time from the start of the first mucosal incision until the end of the lesion's excision. The dissection speed was calculated as follows:
  Short diameter of excised specimen × long diameter of excised specimen × 0.25 × 3.14 / procedure time.^23^
  Postoperative esophageal stricture, perforation, posterior hemorrhage, and pneumonia were noted as ESD‐related adverse events. Posterior hemorrhage was defined as cases requiring emergency endoscopy because of hematemesis or hemorrhage following the completion of ESD, as well as cases wherein hemoglobin levels decreased by ≥2 g/dL.^5^ Pneumonia was defined as cases wherein a postoperative chest radiograph showed pneumonia, or those wherein pneumonia was diagnosed clinically and required antimicrobial therapy.^5^
5. Standard handling of resected specimens.

The macroscopic type was classified as protruding (types 0‐Ip or 0‐Is) or flat (types 0‐IIa, 0‐IIb, or 0‐IIc). Cancer invasion depth was diagnosed according to the Japanese Classification of Esophageal Cancer.^18^ BE was defined as potentially including new muscularis mucosa just under the columnar epithelium. Primary muscularis mucosae were classified as DMM and new muscularis mucosae were classified as superficial muscularis mucosae (SMM). Cancers that extended beyond the basement membrane into the SMM or lamina propria mucosa were classified as lamina propria mucosa, while those that invaded the deepest muscularis mucosa were classified as DMM. The resection margin was classified as follows: pR0, no cancer was pathologically recognized in any resection margin; pR1, cancer was pathologically recognized in any resection margin; and pRX, there was residual cancer in any resection margin that could not be assessed pathologically. All pathological diagnoses were performed by the trained pathology specialist of the facility where the ESD was performed.

### Data collection

The patient, lesion, and treatment characteristics were obtained from the medical records of the participating centers. The analyzed patient data included sex, age, lesion location, BE type, treatment outcomes, and ESD‐related adverse events. The histological lesion features were recorded from the pathology reports. These data included macroscopic appearance, tumor size, depth of invasion, histological type, lymphovascular invasion, and resection margins. Follow‐up patient data were evaluated based on data collected until March 2024. These included local recurrence of metachronous BEA after ESD, as well as metastasis to the lymph nodes and other organs. Local recurrence after ESD was defined as adenocarcinoma adjacent to the scar or arising from the ESD.

### Statistical analyses

Unpaired Student's t‐ and χ^2^‐tests were used for the statistical examination. Continuous variables were expressed as means and standard deviations. The statistical significance levels of any differences identified between pairs of groups were calculated using Student's t‐, Shapiro‐Wilk, or Levine's tests, as appropriate. Statistical significance was set at *p* < 0.05. All statistical analyses were conducted using Statistical Package for the Social Sciences software, version 26 (IBM Corp.).

### Ethical approval

An opt‐out method was used to obtain informed consent from the included patients, as only anonymized retrospective data were used. The study's protocol was approved by the Ethics Committee of Kagoshima University Graduate School of Medical and Dental Sciences, and subsequently by the institutional review board of each of the participating centers (approval number: 230303; approved April 15, 2024). The requirement for written informed consent was waived, owing to the study's retrospective design. The study was conducted in accordance with the principles of the Declaration of Helsinki.

## RESULTS

### Patient characteristics

A total of 283 patients with 287 lesions were included in this study. Their median age was 67 years, and the male‐to‐female ratio was 38:7, as 84.4% were male. A total of 36.7% were obese, with BMIs of ≥25 kg/m^2^. The proportion who were taking antacids at the time of diagnosis was 43.5%, and 21.6% were Hp positive. In terms of background BE, 225 (79.5%) and 58 (20.5%) cases were caused by Short segment BE and long segment BE, respectively. Reflux esophagitis with mucosal damage accounted for 21.2% of the cases, most of which were grade A according to the revised Los Angeles classification. Esophageal hiatus hernia was a complication in 78.1% of the cases (Table 1).

**TABLE 1:** Characteristics of patients and comparison between the early and the late periods.

	Patients with BEA (*n* = 283)	Early period (*n* = 86)	Late period (*n* = 197)	*p*‐value
**Age (years), median ± SD (range)**	67.0 ± 11.7 (38–92)	66.0 ± 12.4	67.4 ± 11.5	0.377
**Sex, male/female**	236/47	71/15	165/32	0.803
**BMI, mean ± SD (range)**	23.9 ± 3.9 (15.1–43.5)	24.0 ± 4.3	23.9 ± 3.8	0.811
**Alcohol, *n* (%)**				
None of chance	128 (45.2)	36 (41.9)	92 (46.7)	0.075
Past	15 (5.3)	5 (5.8)	10 (5.1)	
<21.6 g	33 (11.7)	13 (15.1)	20 (10.2)	
21.6–43.2	73 (25.8)	18 (20.9)	55 (27.9)	
>43.2 g	31 (11.0)	11 (12.8)	20 (10.2)	
Unknown	3 (1.1)	3 (3.5)	0 (0.0)	
**Smoking, *n* (%)**				
None	94 (33.2)	33 (39.8)	63 (31.3)	0.173
Current or past smoker	186 (65.7)	50 (60.2)	138 (68.7)	
Unknown	3 (1.1)	3 (3.5)	0 (0.0)	
**Comorbidity, *n* (%)**				
Cardiac disease	44 (15.5)	13 (15.1)	31 (15.7)	0.895
Respiratory disease	24 (8.5)	6 (7.0)	18 (9.1)	0.549
Cerebrovascular disease	24 (8.5)	5 (5.8)	19 (9.6)	0.287
Diabetes	69 (24.4)	17 (19.8)	52 (26.4)	0.292
**Cancer of other organs, *n* (%)**	54 (19.1)	15 (17.4)	39 (19.8)	0.643
**Antithrombotic drug, *n* (%)**	42 (14.8)	9 (10.5)	33 (16.8)	0.171
**Anti‐acid agent, *n* (%)**				
None	160 (56.5)	49 (57.0)	111 (56.3)	0.003
H2RA	16 (5.7)	6 (7.0)	10 (5.1)	
PPI	76 (26.9)	30 (34.9)	46 (23.4)	
PCAB	31 (11.0)	1 (1.2)	30 (15.2)	
**Hp infection, *n* (%)**				
Uninfected	191 (67.5)	52 (60.5)	139 (70.6)	0.194
Eradicated	43 (15.2)	13 (15.1)	30 (15.2)	
Infected	18 (6.4)	7 (8.1)	11 (5.6)	
Unknown	31 (11)	14 (16.3)	17 (8.6)	
**Barret's esophagus, SSBE/LSBE**	225/58	69/17	156/41	0.841
**Reflux esophagitis with mucosal break, *n* (%)**				
None	223 (78.8)	66 (76.7)	157 (79.7)	0.847
A	47 (16.6)	16 (18.6)	31 (15.7)	
B	8 (2.8)	3 (3.5)	5 (2.5)	
C	5 (1.8)	1 (1.2)	4 (2.0)	
D	0 (0)	0 (0)	0 (0)	
**Hiatal hernia, *n* (%)**	221 (78.1)	71 (82.6)	150 (76.1)	0.230

Abbreviations: BEA, Barrett's esophageal adenocarcinoma; BMI, body mass index; H2RA, histamine type‐2 receptor antagonists; HP, *Helicobacter pylori*; LSBE, long segment Barrett's esophagus; PCAB, potassium competitive acid blockers; PPI, proton pump inhibitors; SD, standard deviation; SSBE, short segment Barrett's esophagus.

**FIGURE 2:**
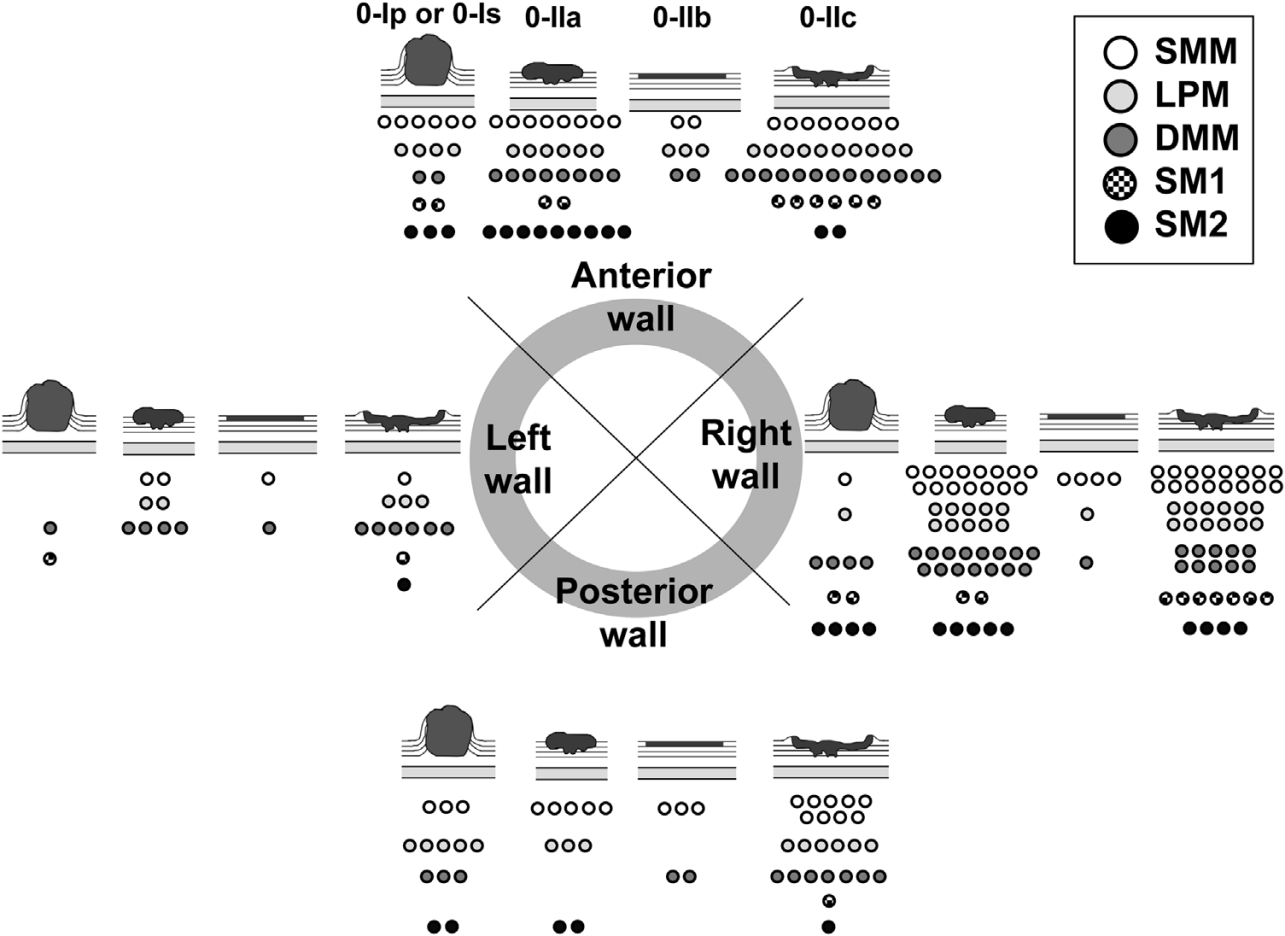
Lesion location, depth of invasion, and microscopic type. SMM, superficial muscularis mucosa; LPM, lamina propria mucosa; DMM, deep muscularis mucosa; SM1, inner third invasion of the submucosa; SM2, middle third invasion of the submucosa.

A total of 72.8% of the lesions were located in the anterior or right wall, and ∼69.3% measured <25% of the circumference. The most common depth of the lesion was SMM, followed by DMM and lamina propria mucosa (28.6%, 27.5%, and 23.0%, respectively). A total of 79.1% of the cases were T1a, with 0‐IIc being the most common macroscopic type (Table 2 and Figure 2).

**TABLE 2:** Characteristics of lesions and comparison between the early and the late periods.

	BEA lesions (*n* = 287)	Early period (*n* = 86)	Late period (*n* = 201)	*p*‐value
**Location, *n* (%)**				
Anterior wall	96 (33.4)	30 (34.9)	65 (33.0)	0.046
Right wall	113 (39.4)	41 (47.7)	72 (35.8)	
Posterior wall	52 (18.1)	9 (10.5)	43 (21.4)	
Left wall	25 (8.7)	5 (5.8)	20 (10.2)	
Whole circumference	1 (0.3)	1 (1.2)	0 (0.0)	
**Circumferentially of lesions, *n* (%)**				
<24%	199 (69.3)	64 (74.4)	135 (67.2)	0.186
25%–49%	64 (22.3)	18 (20.9)	46 (22.9)	
50%–74%	18 (6.3)	2 (2.3)	16 (8.1)	
75%–99%	5 (1.7)	1 (1.2)	4 (2.0)	
100%	1 (0.3)	1 (1.2)	0 (0.0)	
**Macroscopic type, *n* (%)**				
0‐Ip	5 (1.7)	4 (4.7)	1 (0.5)	0.085
0‐Is	39 (13.6)	11 (12.8)	28 (14.2)	
0‐IIa	98 (34.1)	30 (34.9)	68 (33.8)	
0‐IIb	20 (7.0)	3 (3.5)	17 (8.6)	
0‐IIc	125 (43.6)	38 (44.2)	87 (43.3)	
**Depth of lesion, *n* (%)**				
SMM	82 (28.6)	27 (31.4)	55 (27.4)	0.504
LPM	66 (23.0)	16 (18.6)	50 (24.9)	
DMM	79 (27.5)	22 (25.6)	57 (28.4)	
SM1	24 (8.4)	8 (9.3)	16 (8.1)	
SM2	35 (12.2)	12 (14.0)	23 (11.7)	
Others	1 (0.3)	1 (1.2)	0 (0.0)	
**Lymphatic invasion, 0/1**	260/27	76/10	184/17	0.399
**Vascular invasion, 0/1**	274/13	80/6	194/7	0.192
**Horizontal margin, 0/1/x**	267/19/1	78/7/1	186/14/1	0.754
**Vertical margin, 0/1/x**	267/19/1	81/5/0	183/13/1	0.777
**Mean resected length diameter (mm), mean ± SD (range)** ^†^	43 ± 15.5 (11–135)	45.1 ± 17.2	42.2 ± 14.6	0.15
**Mean resected short diameter (mm), mean ± SD (range)** ^†^	32.1 ± 12.2 (6–102)	34.8 ± 14.3	30.9 ± 11.0	0.025
**Mean tumor length diameter (mm), mean ± SD (range)** ^†^	19.2 ± 13.8 (1–108)	19.7 ± 14.2	19.1 ± 13.7	0.705
**Mean tumor short diameter (mm), mean ± SD (range)** ^†^	12.5 ± 8.7 (1–73)	13.2 ± 8.3	12.2 ± 8.9	0.357
**Area of resection specimen (cm^2^) mean ± SD** ^†^	12.1 ± 10.3	14.2 ± 11.6	11.2 ± 9.5	0.022

^†^ Data are shown as mean ± standard deviation (range).

Abbreviations: DMM, deep muscularis mucosa; LPM, lamina propria mucosa; SD, standard deviation; SM1, inner third invasion of the submucosa; SM2, middle third invasion of the submucosa; SMM, superficial muscularis mucosae.

### Treatment outcomes

The treatment outcomes were: en bloc resection rate, 98.6%; complete resection rate, 73.1%; procedure time, 102 min; and dissection speed, 13.6 mm^2^/min. Regarding adverse events, the perforation rate was 0.7%; delayed bleeding rate was 1.8%; postoperative pneumonia was 4.6%; and postoperative esophageal stricture occurred in 3.5% of the cases. Of the 72 patients who underwent noncurative resection, 42 underwent additional surgical resection. Recurrence was observed in eight patients—of whom two experienced metachronous recurrence, two had local recurrence, two had lymph node recurrence, and one experienced recurrence in another organ (Data S1). The mean follow‐up duration was 1306 days, and five of the patients died of their original disease (Table 3).

**TABLE 3:** Outcome and prognosis of endoscopic submucosal dissection.

	Patients with BEA (283 patients 287 lesions)
***En bloc* resection rate**	98.6% (283/287)
**Complete resection rate**	73.1 (211/283)
**Procedure time (min)**	102.0 ± 64.0
**Dissection Speed (mm^2^/min)**	13.6 ± 9.6
**Perforation, *n* (%)**	2 (0.7)
**Delayed bleeding, *n* (%)**	5 (1.8)
**Pneumonia, *n* (%)**	13 (4.6)
**Esophageal stricture after ESD, *n* (%)**	10 (3.5)
**Additional surgery, *n* (%)**	42 (14.8)
**Lymph node metastasis rate after additional surgery, *n* (%)**	7 (16.7)
**Recurrence, *n* (%)**	8 (2.8)
**Recurrence lesion**	
Focal recurrence	4
Lymph node recurrence	1
Other	4
**Prognosis (alive/dead)**	257/26
**Cause of death (primary/other)**	5/21
**Follow‐up period (days), mean ± SD (range)**	1306 ± 1172 (5–4878)

Abbreviations: BEA, Barrett's esophageal adenocarcinoma; ESD, endoscopic submucosal dissection; SD, standard deviation.

### Early versus late period

**FIGURE 3:**
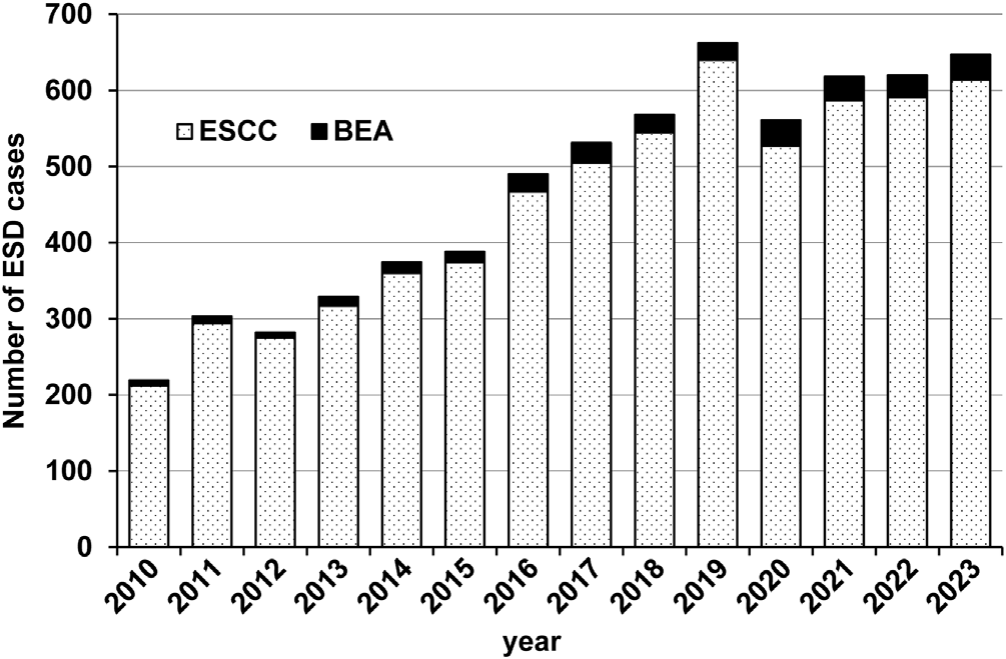
Proportion of BEA cases among the total esophageal ESD cases. BEA, Barrett's esophageal adenocarcinoma; ESSC, esophageal squamous cell carcinoma; ESD, endoscopic submucosal dissection.

The numbers of patients with SCC and BEA who underwent esophageal ESD over the study period are presented in Table 4 and Figure 3. The total number of esophageal ESD cases increased from 2299 over the first 7 years to 4009 over the second. Among these, BEA accounted for a significantly higher percentage as the study progressed—from 3.6% (86/2299) in the early period to 4.7% (197/4009) in the latter one (*p* = 0.034). A comparison of the patient backgrounds between the earlier and later period groups revealed an increase in vonoprazan use (*p* = 0.003; Table 1). The areas of the resected specimens were smaller during the later period vs. the earlier one (Table 2). Regarding treatment results, the procedure time was significantly shorter but the dissection speed remained unchanged. No significant difference was observed concerning the incidence of accidental injuries between the early and late periods (Table 5).

**TABLE 4:** Number of esophageal squamous cell carcinoma and Barrett's esophageal adenocarcinoma (BEA) cases undergoing endoscopic submucosal dissection over time.

	Early period	Late period	Odds ratio	95% CI	*p*‐Value
**BEA, *n***	86	197			
**Esophageal SCC, *n***	2299	4009			
**Total number of esophageal ESD, *n***	2385	4206			
**Percentage of BEA cases, %**	3.6%	4.7%	1.314	1.014–1.701	0.034

Abbreviations: BEA, Barrett's esophageal adenocarcinoma; CI, confidence interval; ESD, endoscopic submucosal dissection; SCC, squamous cell carcinoma.

**TABLE 5:** Comparison of outcome and adverse events of endoscopic submucosal dissection between the early and late periods.

	Early period 86 patients 86 lesions	Late period 197 patients 201 lesions	*p*‐value
***En bloc* resection rate, % (*n*/*n*)**	100% (86/86)	98.0% (197/201)	0.183
**Complete resection rate, % (*n*/*n*)**	74.4% (64/86)	71.1% (147/201)	0.821
**Procedure time (min), mean ± SD**	122.1 ± 81.2	93.2 ± 53.3	<0.001
**Dissection speed (mm^2^/min), mean ± SD**	13.9 ± 13.2	13.4 ± 7.4	0.625
**Perforation, *n* (%)**	0 (0.0)	2 (1.0)	0.348
**Delayed bleeding, *n* (%)**	1 (1.2)	4 (2.0)	0.61
**Pneumonia, *n* (%)**	4 (4.7)	9 (4.6)	0.976
**Esophageal stricture after ESD, *n* (%)**	2 (2.3)	8 (4.1)	0.467

Abbreviations: ESD, endoscopic submucosal dissection; SD, standard deviation.

## DISCUSSION

To the best of our knowledge, this multicenter retrospective study represents the first detailed real‐world investigation of a large number of cases of BEA and endoscopic outcomes in the Kyushu region, which represents the fastest‐aging region in Japan. Our results showed that the incidence of BEA among the patients with early‐stage esophageal cancer who underwent ESD increased in the region between 2010 and 2023.

SCC represents the most common esophageal cancer in Japan. However, BEA is more common in Western countries, including the United States.^24^ Epidemiological data concerning EGJ junction cancers in Asia are extremely scarce in the literature.^25^ In Japan, the incidence of BEA has been reported to be increasing^11^, ^26^; however, these reports are mostly from databases,^11^, ^27^, ^28^ and the results vary depending on the database. Data concerning whether BEA is actually increasing in real‐world endoscopic practices therefore remain unknown. In this study, despite the increase in esophageal ESD cases that we observed, the ratio of ESD to BEA increased—indicating a higher proportion of patients with BEA. This may be due to an increase in the number of patients with gastroesophageal reflux disease,^8^ and BE,^26^ as well as a decrease in the rate of Hp infection.^9^ We found no difference regarding the increase in BMI, which has been reported to be related to gastroesophageal reflux disease or Hp infection, between the early and late periods. There was also no change in the proportion of patients with reflux esophagitis and mucosal breaks, or the rate of hiatal hernia. This may be due to several reasons: (1) the development of IEE and similar high‐resolution endoscopic techniques,^1^, ^2^, ^3^, ^4^ (2) increased dissemination of information concerning BEA among Japanese endoscopists, and (3) an actual increase in the number of patients with BEA. To clarify whether the observed increase in BEA cases was due to the development of endoscopic technology and knowledge dissemination among endoscopists, we examined the data using linear regression analysis by year. This revealed that the number of endoscopic treatments over the 14‐year study period had increased significantly (data not shown). As opportunities for endoscopists to treat BEA are increasing, it has become increasingly necessary for them to have a good understanding of endoscopic BEA imaging.

This study included patients who were eligible for ESD and had pathologically confirmed BEA. This allowed for a clear differentiation from gastric cancer arising at the EGJ, as well as an accurate evaluation of morbidity. Furthermore, it is difficult to determine whether advanced EGJ adenocarcinoma originates from the esophagus or stomach. This is because it is impossible to confirm its origins between the muscularis mucosa vs. proper esophageal glands, which are pathologically characteristic of BEA. Therefore, unlike the case for surgically‐resected lesions, accurate data concerning BEA occurrence cannot be gleaned from lesions resected via ESD.

SSBE carcinogenesis accounted for a high proportion of BEA during both the early and late periods of this study, which is consistent with previous reports from Japan.^29^ The increased use of vonoprazan over the study's later period reflects the context of the study.

Regarding the ESD results for BEA, the resection area was significantly smaller during the later period of the study vs. the early one. The dissection speed remained constant, but the treatment time was significantly shorter. We hypothesized that this was because endoscopic imaging for BEA became a more widespread practice among endoscopists over the study period, leading to the detection of smaller lesions.

The complication rate of ESD for treating BEA was lower in this study compared to what has previously been reported. In Japan, gastric and esophageal ESD began to be covered by the country's national health insurance program in 2004^30^ and 2008,^18^ respectively.

This study was subject to several limitations key limitations worth noting. Although it was a multicenter study, it was retrospective in nature. Second, the resected lesions were diagnosed by different pathologists at each facility. Third, the background and treatment outcomes of the patients with esophageal SCC who underwent ESD were not examined. Fourth, the study did not include patients who underwent surgery to treat early‐stage BEA. Fifth, there was no uniformity regarding the levels of experience among the ESD practitioners, the devices used, or the ESD methods employed. Sixth, BEA cases that were treated via EMR were excluded from this study. Future studies should therefore consider addressing these shortcomings.

This study demonstrated that the proportion of BEA cases among those of esophageal ESD has increased over recent years in Japan's Kyushu region. However, as with conventional esophageal SCC, the treatment outcomes of ESD for BEA are good; therefore, we believe that early diagnosis of esophageal tumors will help improve patient quality of life.

## CONFLICT OF INTEREST STATEMENT

None.

## ETHICS STATEMENT

Approval of the research protocol by an institutional review board: The study protocol was approved by the Ethics Committee of Kagoshima University Graduate School of Medical and Dental Sciences, as well as subsequently by each institutional review board of the other participating centers (approval number: 230303, approved April 15, 2024).

## PATIENT CONSENT STATEMENT

An opt‐out method was used to obtain informed consent from the included patients, as only anonymized retrospective data were used. The requirement for written informed consent was waived, owing to the study's retrospective design. The study was conducted in accordance with the principles of the Declaration of Helsinki.

## CLINICAL TRIAL REGISTRATION

Not applicable.

## Supporting information




**DATA S1** Clinical and pathological characteristics of patients with recurrence.

## Data Availability

The data that support the findings of this study are available from the corresponding author upon reasonable request.
