# Supplementary material for: Clinical backgrounds and outcomes of patients with Barrett's esophageal adenocarcinoma treated via endoscopic submucosal dissection in Kyushu, Japan: A retrospective multicenter cohort study
Source: DEN Open. 2025 May 5;6(1):e70102. doi: 10.1002/deo2.70102 (PMC12052473; doi:10.1002/deo2.70102)
Supplement: Supplementary file 1 — DATA S1 Clinical and pathological characteristics of patients with recurrence. [file DEO2-6-e70102-s001.docx]

**Supplemental Data;** Clinical and pathological characteristics of patients with recurrence

| **Age/Sex** | **Macroscopic Type** | **Histological type** | **Depth of the lesion** | **En block resection** | **R0 resection** | **Lymphatic invasion** | **Vascular invasion** | **Horizontal mergin** | **Verticall mergin** | **Lesion size (mm)** | **Time until recurrence (Month)** | **Recurrence pattern** | **Treatment for recurrence** |
| --- | --- | --- | --- | --- | --- | --- | --- | --- | --- | --- | --- | --- | --- |
| 82/M | Ip | Well differenciated | LPM | + | + | - | - | HM0 | VM0 | 23×20 | 12 | Metachronous | Endoscopic resection |
| 79/M | IIb | Well differenciated | SMM | + | - | - | - | HM1 | VM0 | 81×20 | 31 | Focal | Endoscopic resection |
| 83/M | Is | Well differenciated | SMM | + | + | - | - | HM0 | VM0 | 20×18 | 10 | Metachronous | Endoscopic resection |
| 81/M | IIa | Well differenciated | SM1 | + | - | - | - | HM0 | VM0 | 45×42 | 9 | Focal | Endoscopic resection |
| 85/M | Is | Well differenciated | SM1 | + | - | + | - | HM0 | VM0 | 20×19 | 16 | Lymph node | Suregery |
| 61/M | IIa | Well differenciated | SM2 | - | - | + | + | HM1 | VM1 | unknown | 24 | Lymph node | Chemotherapy |
| 75/M | IIc | Mixed undifferenciated | SM2 | + | - | - | - | HM0 | VM1 | 32×21 | 34 | Other organs  (Bladder,Bone) | Chemotherapy |
| 73/M | IIa | Well differenciated | SM2 | - | - | - | - | HM0 | VM1 | 35×22 | 33 | Lymph node | Best supportive care |
